# Exoskeletons With Virtual Reality, Augmented Reality, and Gamification for Stroke Patients’ Rehabilitation: Systematic Review

**DOI:** 10.2196/12010

**Published:** 2019-09-08

**Authors:** Omar Mubin, Fady Alnajjar, Nalini Jishtu, Belal Alsinglawi, Abdullah Al Mahmud

**Affiliations:** 1 School of Computing, Engineering and Mathematics Western Sydney University Rydalmere Australia; 2 College of Information Technology United Arab Emirates University Al Ain, Abu Dhabi United Arab Emirates; 3 Swinburne University of Technology Melbourne Australia

**Keywords:** stroke, robot, exoskeleton, virtual reality, augmented reality, gamification, rehabilitation

## Abstract

**Background:**

Robot-assisted therapy has become a promising technology in the field of rehabilitation for poststroke patients with motor disorders. Motivation during the rehabilitation process is a top priority for most stroke survivors. With current advancements in technology there has been the introduction of virtual reality (VR), augmented reality (AR), customizable games, or a combination thereof, that aid robotic therapy in retaining, or increasing the interests of, patients so they keep performing their exercises. However, there are gaps in the evidence regarding the transition from clinical rehabilitation to home-based therapy which calls for an updated synthesis of the literature that showcases this trend. The present review proposes a categorization of these studies according to technologies used, and details research in both upper limb and lower limb applications.

**Objective:**

The goal of this work was to review the practices and technologies implemented in the rehabilitation of poststroke patients. It aims to assess the effectiveness of exoskeleton robotics in conjunction with any of the three technologies (VR, AR, or gamification) in improving activity and participation in poststroke survivors.

**Methods:**

A systematic search of the literature on exoskeleton robotics applied with any of the three technologies of interest (VR, AR, or gamification) was performed in the following databases: MEDLINE, EMBASE, Science Direct & The Cochrane Library. Exoskeleton-based studies that did not include any VR, AR or gamification elements were excluded, but publications from the years 2010 to 2017 were included. Results in the form of improvements in the patients’ condition were also recorded and taken into consideration in determining the effectiveness of any of the therapies on the patients.

**Results:**

Thirty studies were identified based on the inclusion criteria, and this included randomized controlled trials as well as exploratory research pieces. There were a total of about 385 participants across the various studies. The use of technologies such as VR-, AR-, or gamification-based exoskeletons could fill the transition from the clinic to a home-based setting. Our analysis showed that there were general improvements in the motor function of patients using the novel interfacing techniques with exoskeletons. This categorization of studies helps with understanding the scope of rehabilitation therapies that can be successfully arranged for home-based rehabilitation.

**Conclusions:**

Future studies are necessary to explore various types of customizable games required to retain or increase the motivation of patients going through the individual therapies.

## Introduction

### Background

Stroke refers to a sudden, often catastrophic neurological event that can lead to long-term adult disability. The American Heart Association (AHA) is responsible for providing up-to-date statistics related to heart disease and stroke. According to Benjamin et al [1], the AHA released a 2017 statistics report on heart disease and stroke that stated that approximately 795,000 stroke episodes occur in the US each year. With current advancements in medical technology there has been a decrease in the rate of stroke incidents, but it can still cause paralysis and muscle weakness. Such impairments can result in motor deficits that disturb a stroke survivor's capacity to live independently.

There are several reasons for stroke occurrence, which could be related to an increased risk of a collection of symptoms caused by disorders affecting the brain (eg, dementia) [2]. Various rehabilitation techniques have been used in the area of rehabilitation-based interactive technology to assist patients in recovering from impairments, and those techniques come under the umbrella of conventional therapy, exoskeleton or robot-aided therapy, virtual reality (VR) or augmented reality (AR) therapy, games-based therapy, or a combination of any of these. These forms of therapy can be done either in the clinic or in an in-home setting. In addition to these, there is a new technology known as telerehabilitation [3] that leverages the use of VR in home settings by providing patients access to real-time rehabilitation services through the internet while they sit at home.

One of the most effective techniques is robot-aided therapy, which has been gradually increasing in use primarily because patients may consider traditional rehabilitation therapy to be tiring and exhaustive. This may decrease their motivation and cohesion to the treatment, thus resulting in only minor improvement in the health of poststroke patients [4-6]. Various experimental evidence suggests that robot-assisted (or exoskeleton) rehabilitation has been effective in keeping patients motivated and interested in treatment for both upper or lower limb impairments [7,8]. With advancements in technology, there has also been an uptake of VR, AR, and Gamification for the purposes of rehabilitation [9], along with robotic rehabilitation [10,11], primarily to increase engagement, immersion and motivation on behalf of the patient. Both Colombo et al and Alankus et al [12,13] concluded and showed the positive effect of exoskeleton robots and games in poststroke rehabilitation. Wearable devices such as exoskeletons can also relay real-time feedback for any VR-based interactions [14].

Apart from these studies, Housman et al [15] showed user satisfaction survey results in which 90% of participants agreed to the fact that robot- or games-assisted therapies were less confusing, and improvements were very easy to track compared to traditional or conventional therapies. Further, it is thought that gamification can increase repetition, engagement, and range of care within the context of rehabilitation [16,17]. Games are not only useful for the field of rehabilitation, but they are also considered to be highly impactful and relevant in other medical and health fields. Russoniello et al [18] conducted a randomized controlled trial (RCT) study in which the effects of video games on stress-related disorders were tested, with the conclusion being that games were beneficial for their prevention and treatment. In another study, children who had cerebral palsy made use of a game (EyeToy) which was able to improve their upper extremity functions over time [19].

### Virtual Reality, Augmented Reality and Gamification

VR is a virtual form of a real entity, object or environment. According to Schultheis et al [20], VR can be regarded as an enhanced version of human-computer interaction (HCI) in which the human interacts with a three-dimensional (3D) interface and is immersed in a synthetic environment comprised of digital objects. Various devices, such as earphones and head-mounted displays (HMDs), are used to support this form of technology. VR has already become popular in the fields of science, music, education and training, and healthcare, but in areas such as poststroke rehabilitation it has been an immense benefit. For example, Katz et al [21] described the effectiveness of VR in treating poststroke patients through their street program. In this study, the patients were suffering from Unilateral Spatial Neglect (USN), which happens because of right hemisphere—caused stroke. VR can provide the opportunity to create and customize a patient’s training based on their interests. This could increase their motivation to continue training and increase their attention during their sessions, both of which are essential factors for effective rehabilitation.

As mentioned above, traditional methods of rehabilitation might lead to a patient’s loss of interest in their therapy, as it often involves daily repetitive tasks. VR encourages patients to participate in their therapy by either incorporating games in the form of exercises or through other interactive means. With the current state of VR in rehabilitation services, a new form of therapy has gradually emerged that is known as Virtual Rehabilitation [22]. Virtual rehabilitation is defined as the ability of VR to provide therapy to patients using its hardware and simulation. Apart from a definition, Burdea also lists classifications and taxonomy for Virtual Rehabilitation [22]. Classification is done based on the area of study, the rehabilitation protocol, or the availability of a therapeutic team for the patient. Hardware used in VR is multipurpose and can be used for different patients suffering from different types of strokes (eg, a hand glove can be used to do strengthening exercises as well as other motor improvement exercises.) Therefore, VR provides an interactive and motivational environment, where patients feel encouraged to participate in clinical or home-based trials.

We must also consider the advancements in VR technology in recent years by the inclusion of sixth and seventh generation gaming systems, which include various popular systems such as the Xbox 360 Kinect and the Nintendo Wii. Yates et al [23] discussed various commercial gaming systems and gave extensive information regarding the features of these VR systems. When more realism (such as through the inclusion of tangible or physical objects in the virtual world) is added to VR, it gives rise to a new technology called AR. The user feels more realism as they receive more control over virtual objects by interacting with real objects. The virtual view of the world, or an environment, is superimposed in the real world, so therefore VR and AR lie at the opposite ends of a reality-virtuality spectrum.

Slowly, AR is also gaining traction in the field of rehabilitation. According to Khademi et al [24], when a haptic device was used with AR in an experiment, there were improvements in hand stiffness that proved the potential of a haptic AR rehab system. In another study, Mousavi et al [25] trained and assessed subjects side by side with the help of multiple groups. One group used AR while the other one used traditional HCI via a personal computer and a mouse. The results of this study showed increased motor movements in the group using the AR technology as compared to the traditional means of interaction.

In addition to these two technologies (VR and AR), video games are often used in rehabilitation services these days as they play an essential part in encouraging patients to participate in therapeutic exercises. It should be noted that VR- or AR-interaction can be nongamified or nonplayful, which is why we prefer to delineate them. Games are used with specific hardware depending on the physical condition of the patients, and various game attributes are considered while developing games for rehabilitation purposes. There are several types of games used in rehabilitation services, such as two-dimensional (2D), 3D, VR and AR games, and other natural user interfaces such as Wii, PlayStation, Wii Balance, Xbox, and Kinect. Alankus et al [13] developed games for stroke-affected patients and identified three important attributes in this space: social context (multiplayer versus single player), motion type (single-muscle motion versus multiple-muscle motion) and cognitive challenge (easy versus difficult). Audio-visual cues and performance-related online information was also provided to patients as another means of boosting their motivation.

It should be noted that most of the games used in rehabilitation are commercial, off-the-shelf games. In a study conducted by Acosta et al [26], the feasibility of using the Nintendo Wii was assessed in a group of 20 patients, and it was concluded that use of computing gaming devices might be a benefit for rehabilitation. Burke et al [27] discussed various games (eg, *Rabbit Chase*, *Bubble Trouble* and *Arrow Attack*), and identified game design principles which were significant for upper limb stroke rehabilitation. However, the aim of our paper is to investigate and review the coupling of such gaming elements, or virtual reality, with an exoskeleton or robotic device.

Exoskeleton means an extension to the (human) skeleton, but in simplistic terms, some researchers have defined an exoskeleton as any transparent device that a user or patient may wear or attach upon themselves and that extends their natural motor capabilities by determining their intent [28]. They are popular for enhancing human strength and speed via their internal components, which is a composition of electric motors, levers, and hydraulics. There are many exoskeletons available, like Amadeo, HandCARE, ARMin IV, and CyberGlove, that are used to assist patients in participating in rehabilitation sessions.

To summarize, in our review we intended to ascertain the possible interactions of rehabilitation robotics or exoskeletons with AR or VR (forming the two major components considered within our review, that is, the hardware and the interfacing technology), that is, to use the intermediate interfaces employed as a means of supplementing the rehabilitation process using exoskeleton-based hardware. Therefore, we set out to perform an exploratory review of the field of rehabilitation robotics with an additive aspect of technical scope, focusing on solutions and prototypes in the area of exoskeletons that interfaced with software mediums. Our methodology focused on the common approach carried out when doing systematic reviews; however, our analysis and reflections were mainly based on qualitative grouping and meta-synthesis, due to the preliminary nature of our work and the heterogeneous and multidisciplinary character of our considered papers.

To further motivate our approach, work and research objectives, we scanned the literature to extract review articles like ours, and two recent studies emerged. The first focused primarily on the prospect of exoskeletons for stroke rehabilitation [29] and the second discussed the possibility of VR-based interactions for rehabilitation [30]. We essentially combined the two and investigated what would happen when both hardware-based rehabilitation aids and software interfaces depicting virtual reality or gaming mechanisms were merged.

Therefore, to conclude, the aim of this review was to examine the potential and latest trends in the area of exoskeleton- or robotic-aided therapy in combination with VR, AR, or gamification for the improvement of motor function for poststroke patients. Specifically, we aimed to determine: (1) if such a coupled approach or setting could provide positive outcomes for patients; (2) trends and popular configurations across both types of exoskeletons and software mediums; and (3) future challenges in the field of exoskeleton-based HCI therapy.

## Methods

### Databases Searched and Search Terms Used

We conducted this review according to the Preferred Reporting Items for Systematic Reviews and Meta-Analyses (PRISMA) guidelines [31]. The following databases were searched for relevant studies: MEDLINE, EMBASE, Science Direct and The Cochrane Library, and studies conducted between the years 2010 to 2017 were included. An electronic search of the literature was performed using search terms such as “post-stroke rehabilitation, exoskeleton, robotic device, virtual reality, or augmented reality, or gamification”.

### Inclusion and Exclusion Criteria

Inclusion criteria were as follows: (1) experimental, explorative or RCT studies on poststroke rehabilitation, (2) VR, AR, or gamification visual feedback, (3) stroke-affected patients; and (4) use of an exoskeleton or robotic device. As per our interpretation for this review, a VR or AR environment in the field of robot-aided rehabilitation is a replica of the real-world environment that is achieved after using hardware devices and a wearable exoskeleton device in liaison with each other.

The exclusion criteria for our study were: (1) studies done without the use of any robotic device or exoskeleton; (2) studies with nonvirtual or nonaugmented environments, or an absence of games; and (3) publications or articles in languages other than English. As mentioned before, due to the heterogenous nature of the collated studies, data was synthesized qualitatively.

## Results

### Search Results

**Figure 1:**
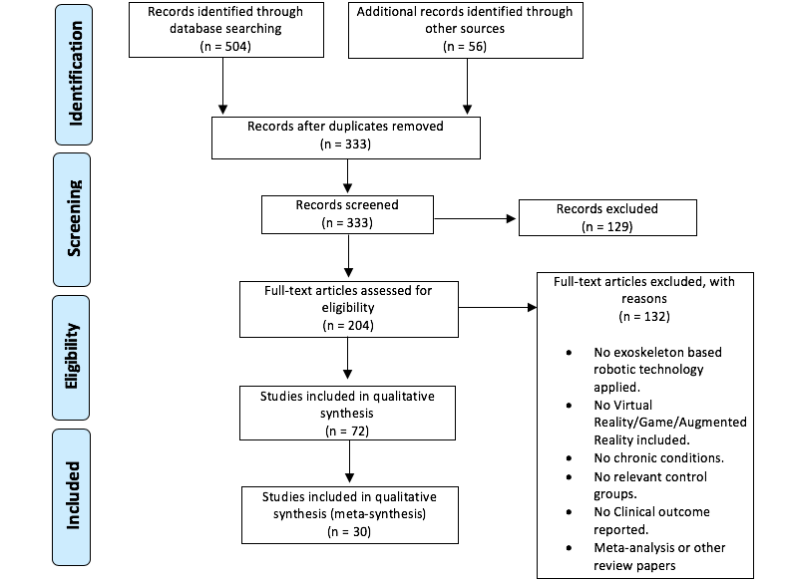
Selection of articles for review.

A total of 504 articles were identified from electronic searches and a total of 56 were identified through reference searches or other sources. We excluded 129 citations which were only at title and abstract stage, resulting in 204 full-text articles. Of these, 132 citations were excluded at the full-text stage, with reasons mentioned in Figure 1 as per the exclusion criteria for this paper. 30 studies reported across 30 publications were identified for inclusion in this review.

### Overview of Included Papers

All articles published in English and in the years 2010 to 2017 were included. Four studies were published in the year 2010 and one in the year 2017, with size of the samples involved in the studies ranging from 1-47 participants per study; however, 13 studies had less than 10 participants. In two of the studies, healthy users of the devices were used as controls to compare the improvements of the patients [32,33]. Apart from this, one clinical study [34] was performed with just one patient who attended six sessions three times a week for two weeks, where it was found that the VR-based system resulted in effective upper-limb rehabilitation for this patient.

The average time poststroke for the participants involved in these studies, as mentioned in the articles, varied from less than six months to several years. Four studies did not mention the poststroke period at all [32,33,35-37], while three studies (10%) were held during the subacute (less than three months poststroke) stage [38-40]. All the other papers in our sample carried out their studies during the chronic stage (greater than three months poststroke). Most of the studies (28/30) considered designing rehabilitation therapies for upper limb while only two studies involved lower limb. Several measurement assessment scales were used in the studies that were used to assess improvements in motor functions which we then analyzed. Some of the scales included were the Chedoke-McMaster Stroke Assessment (CMSA), the Fugl-Meyer Motor Assessment (FMA), the Wolf Motor Function Test (WMFT), and the Modified Ashworth Scale (MAS), among others. An overview of some of the criteria used is provided as a summarized table below (see Table 1), whereas a detailed overview of the entire dataset is provided in Multimedia Appendix 1. The entire sample of 30 studies is also available in the reference list [26,32-36,38-61].

**Table 1:** Overview of some of the criteria of our review and their associated frequencies (N=30).

Criteria		Relative frequency, n
**Limb Type**		
	Upper limb	28
	Lower limb	2
**Device**		
	Armin	2
	Armeo	2
	Bi-Manu	2
	Other	24
**Degrees of Freedom**		
	<10	15
	>10	1
	Not mentioned	14
**Setting**		
	Clinic	25
	Home	4
	Both	1
**Interaction Type**		
	Games	15
	Virtual Reality	15
**Sample Size**		
	<10	14
	>10	16

## Discussion

### Key Findings

The studies were categorized into different fields, and of all 30 studies half of them used VR therapy while the other half used some gaming concepts. The studies that involved the use of an exoskeleton or robotic device along with VR, AR, or gamification were within the inclusion criteria of this review, so thirty exoskeletons or robotic devices were included. Of these devices, three exoskeletons emerged as slightly more popular in use: ARMin, Bi-Manu Track and ArmeoSpring. These devices each had repeated use in two studies while 24 studies made use of entirely different exoskeletons or robotic devices.

Klamroth-Marganska [41] made use of the ARMin exoskeleton in a 3D workspace with 7 degrees of freedom (DOF) for arm motor impairment in their study. This study was carried out among 38 poststroke patients who attended a total of 24 sessions (45 min/session) where they used VR Games that had their difficulty level adjusted by the therapist. This study resulted in improvement in the affected arm that was trained using ARMin, and audio-visual feedback was also provided to the patients through the VR games to elevate their motivation. Another lab-based empirical study [32] used the fourth version of the ARMin exoskeleton for 30 healthy and 8 impaired subjects, all of who played games with varying difficulty levels during the practice round. After that, feedback was taken from the participants using questionnaires. That study concluded that stroke-affected subjects were more interested in playing multiplayer games as compared to single player, as that allowed them to interact with peers or partners (dependent on the personality traits of the participants).

Another exoskeleton device, Armeo Spring, was used in two other papers selected for analysis [42,43]. In the Grimm et al study, an Armeo Spring device with 7 DOF was used for a clinical study involving five subjects who attended 20 sessions of therapy over four weeks (20 min/session). A VR interface was used with the exoskeleton and the difficulty level of the exercise was adjusted as per a patient’s performance, with a provision for feedback on movement quality. Improvements in kinematic parameters were observed, thus making this particular VR-exoskeleton setup an effective combination for poststroke rehabilitation. In the Gijbels et al article, the Armeo exoskeleton was used with VR-based, nongamified learning (domestic cleaning tasks) for 10 subjects performing exercises three times per week, for a total of eight weeks. Each session lasted for 30 minutes, and auditory-visual performance feedback was provided both before and after the practice. The main outcome of this study was that functional gains in motor movement were reached at the end of the two-month study period, even for patients with high levels of disability.

In the year 2011, several studies were published that made use of exoskeletons with other technologies, and here we summarize a few as case studies. Lambercy et al [44] had a study of 13 poststroke participants using HapticKnob and games which resulted in improvements in their hand and arm motor functions, while Acosta et al [26] used 3D arm coordination training alongside video games and concluded that the duo would be useful for stroke rehabilitation. Similarly, two other studies by da Silva et al and Stein et al [40,62] made use of Data Gloves and Amadeo alongside VR and games, which led to improvements in multiple measures of motor performance in the participants involved in the study. In addition, Bi-Manu-Track with games and Robotic Upper Extremity Repetitive Trainer (RUPERT) with VR were used for both clinical and home-based rehabilitation such as in [53]. In this study, out of the two patients the first showed improvement in movement smoothness on targets while the second did not experience any ascending or descending trend in smoothness.

The studies mentioned so far mostly involved one part of the human body (arm), but Connelly et al [35] discussed hand improvements in which the PneuGlove with 6 DOF (Servomotor actuator) was used in a clinical study that engaged 14 patients for six weeks (60 min/session). An HMD was used to measure haptic feedback in the study, and as a result, a great increase in FMA scores were achieved. In a different study, a home-based trial was done on hand motor function improvements [45] wherein Hand Mentor Pro (HMP) was used alongside video games. From this study, visual biofeedback about the quality and quantity of wrist movements was attained, which resulted in improvements in ARAT scores. In a more recent study, Khor et al [46] discussed the improvements in a 30 min, robot-assisted study for 7 participants who actively took part in clinical and home-based rehab therapy, and who showed improvements in both hand and arm functions. This therapy was assisted by the CR2-Haptic device alongside a VR game, and it was reported that all subjects were comfortable with the therapy. The study also reported on the low cost of its hardware due to a reduced number of sensors and actuators, but this had the negative effect of lowering the customization and scalability of the exoskeleton.

We noticed that there was less academic literature for lower limb rehabilitation compared to upper limb rehabilitation, but our review still included two studies that involved lower limb rehabilitation. Forrester et al and Mirelman et al [47,63] described the effectiveness of the use of the exoskeletons Anklebot (3 DOF) and Rutgers Ankle Rehabilitation System (RARS) (6 DOF), alongside VR and video games, for ankle and foot rehabilitation. Improvements in walking velocity and paretic ankle motor control, as well as an increase in peak plantarflexion moment and in ankle power generation, were observed. Through further snowballing searches after the primary search, we also located two additional studies that employed the Lokomat exoskeleton for leg rehabilitation [64,65]. Both of these studies utilized VR as their key interfacing medium, but the former was a study with adults where a racing game was the main object of interest, while the second was a study with children where games such as soccer were incorporated. Both studies reported generally increased levels of engagement from the participants and thus further outlined the potential of robot-aided rehabilitation for lower limbs using VR. An interesting observation was the absence of AR-based systems for stroke rehabilitation in our sample. The requirement of additional hardware over exoskeletons and real-time tracking might be a deterrent. With the current advancements in AR systems (such as HoloLens), we would expect their application in clinical and medical settings to grow.

### Future Challenges in the Field of Rehabilitation

Although positive results and improvements in motor function were observed in most of the studies, the results from this systematic review also depict that most rehab services are carried out in groups in clinics while home-based rehab is rarely attempted using the current configuration of interactive technologies. Group therapies in a clinical or lab setting are done so that patients feel motivated by collaborating with, or competing against, each other. In home-based rehabilitation, it is possible that patients might feel overwhelmed or isolated with the advanced forms of technology necessary for their therapy. In this case, the technology and the therapy sessions need to be designed in a way such that patients feel motivated and confident during home-based rehabilitation sessions as well (such as through online-tailored gaming). Thus, game-based rehabilitation can play a key role and provide a suitable interfacing medium for VR or AR, with 10 of our sample of 30 papers associating gaming with virtual reality. Use of customized games should be encouraged so that games are designed to keep in mind a particular target user, which could drive motivation in those people who play these specialized games at home as a part of their rehabilitation process. Articles in our sample indicated the key considerations that researchers must focus on while designing games for rehabilitation (also known as serious games), with key elements of discussion including: whether the play is meaningful, if engagement or motivation is retained, the difficulty level of the game, the role of customization, the range and type of feedback acquired, and the overall usability of the gameplay. Lastly, the interaction technique used is also a key consideration in game-based rehabilitation, with what gestures result in what game event easily being dictated by the motor movements required (such as whether the game interaction will involve grasping, pinching or linear limb movements).

However, there are some limitations to this review. For example, different types of assessment scale and quality of collected data were used, which makes it difficult to compare the outcomes and results accurately or quantitatively against each other. In addition, some articles could have been missed in the review due to very specific search criteria.

### Conclusion

This review was carried out to collect data from different clinical trials and then to categorize and explore them to find the effectiveness of VR, AR, or gamification when used in combination with an exoskeleton or robotic device for the rehabilitation of poststroke patients. It was found that very little work is done to make use of these technologies for rehabilitation of lower limbs when compared to upper limbs, and that there are a wide variety of exoskeleton-based devices currently in use. Apart from this, the review also states that these exoskeleton-based devices are rarely available for home-based trials. This shows that there is a considerable gap in the transition of rehabilitation services from a clinical environment to a home-based setting. Future work should focus on the successful application of VR, AR, or gamification technology to engage poststroke patients in rehabilitation therapies done at their homes. In addition, commercial, off-the-shelf games may be deployed easily, but efforts must be dedicated to designing games for rehabilitation to keep in mind the user and allow for customization to facilitate their motivation.

## Appendix

Multimedia Appendix 1 Detailed overview of our dataset of 30 papers.

## Abbreviations

2D: two-dimensional
3D: three-dimensional
AHA: American Heart Association
AR: augmented reality
CMSA: Chedoke-McMaster Stroke Assessment
DOF: degrees of freedom
FMA: Fugl-Meyer Motor Assessment
HCI: human-computer interaction
HMD: head-mounted display
HMP: Hand Mentor Pro
MAS: Modified Ashworth Scale
MRC: Medical Research Council
PRISMA: Preferred Reporting Items for Systematic Reviews and Meta-Analyses
RARS: Rutgers Ankle Rehabilitation System
RCT: randomized controlled trial
RUPERT: Robotic Upper Extremity Repetitive Trainer
USN: Unilateral Spatial Neglect
VR: virtual reality
WMFT: Wolf Motor Function Test
