# Supplementary material for: Exoskeletons With Virtual Reality, Augmented Reality, and Gamification for Stroke Patients’ Rehabilitation: Systematic Review
Source: JMIR Rehabil Assist Technol. 2019 Sep 8;6(2):e12010. doi: 10.2196/12010 (PMC6779025; doi:10.2196/12010)
Supplement: Multimedia Appendix 1 [file rehab_v6i2e12010_app1.pdf]

Appendix A: Table 1

Article Name + Article Web Link	Upper limb / or lower limb	Severity	Assessment scale	Robotic / Exoskeleton Technology (Device)	DOF	Actuator type	Data source / Control input	Of-the-shelf (commercial) / OR Prototype & type model of device if commercial product	Exoskeleton Function / Wearable Exoskeleton placement	Clinical trail / or Home - based rehabilitation	Virtual reality / Augmented Rehabilitation Intervention ? Yes/No	Sample Size	Duration of Exoskeleton VR Rehabilitation program	Time of each VR - AR- GM task/session	Rehabilitation game difficulty level	Real-time feedback - Game	Feedback	Results/improvements
(Lamberc y et al., 2011)	Upper limb	597 .5 ± 294 .1 days	Fugl-Meyer Motor Assessment (FMA)	Haptic Knob (end-effector device)	2	Motor	forearm and hand	Commercial	arm and hand function	Clinical	Games	13	18 hours	60 minutes	Not mentioned	A score of the game was provided as a feedback to the patient, which was then used to adjust the difficulty level of the game.	Image and game are used during study in order to calculate score based on the timing and precision of the task	Thirteen subjects successfully completed robot-assisted therapy, with significantly improved hand and arm motor functions (average 3.00 points increase on the FM and 4.55 on the MI). Also, it was concluded that as distal training in a functional way could benefit the whole arm so there would not be a need to do whole arm training in robot-assisted neurorehabilitation.
(Klamroth-Margans ka et al., 2014)	Upper limb	52± 44 (7 – 171)	Fugl-Meyer Motor Assessment (FMA)	ARMin (in 3D workspace)	7	Motor	Arm motor impairment	Commercial	Arm and Shoulder	Clinical	Virtual Reality (Games)	38	24 sessions	45 minutes	Adjusted by therapist	Not mentioned	Audio - Visual feedback was provided to patients to increase motivation	Robotic training of the affected arm with ARMin was found to be more effective than conventional therapy in terms of motor function (FMA-UE: F = 4·1, p = 0·041, mean difference: 0·78 points, confidence interval [0·03 - 1·53]).
(Khor et al., 2017)	Upper limb	7.9 ± 6.6 months	Fugl-Meyer Motor Assessment (FMA)	proved to b	1	1 actuator	forearm and wrist	Commercial	arm and hand function	Clinical & Home	VR Game	7	30	30min robot-assisted	Not mentioned	VR game provided during the robotic therapy have helped to engage users in a multi-sensory simulated environment that included real-time visual, auditory and tactile feedback	VR game provided during the robotic therapy have helped to engage users in a multi-sensory simulated environment that included real-time visual, auditory and tactile feedback	Improvement of active range of motion was detected in both pronation-supination (75.59%, p = 0.018) and wrist flexion-extension (56.12%, p = 0.018) after the training

(Acosta et al., 2011)	Upper limb	2-15years	Fugl-Meyer Motor Assessment (FMA)	Arm Coordination Training 3D (ACT3D) system	6	Motor	Shoulder	Commercial	Shoulder	Clinical	Video Game	7	56	3 minutes + 1 minutes rest	Difficulty level was based on the requirement for reaching movements of the game.	Game feedback was provided to the patient in the form of an avatar.	Visual feedback on reaching performance through Air Hockey 3D game is given to impaired people/auditory feedback (buzzing noise) was also provided	Reaching distances achieved with the avatar feedback were greater on average than those achieved with Air Hockey 3D across conditions. Therefore results showed that video games and robotic systems that target specific impairments could be developed that would be useful for stroke rehabilitation.
(Wolf et al., 2015)	Upper limb	less than 6 months	Fugl-Meyer Motor Assessment (FMA)	Hand Mentor Pro (HMP)	Not mentioned	Motor	Hand	Commercial	Hand	Home	Video Game	47	40 days (120 hours)	3 hrs/day	Same throughout the therapy and patients were given training	Not mentioned	HMP used a pneumatic artificial muscle to facilitate movement about the wrist and fingers while providing visual biofeedback about the quality and quantity of wrist movements.	Significant improvements were found in a group of patients showing an increase in ARAT scores and WMFT tasks.
(Ueki et al., 2012)	Upper limb	Not provided	Torque measurement device	Hand Motion Assist Robot (Exoskeleton)	18	Servo motor	Hand (thumb and finger)	Commercial	Hand	Clinical	Virtual Reality	6 healthy and 6 stroke patients	-	20 min	Changed according to movable range	Not mentioned	Scores are recorded, and the GUI showed the three scores (today, best, and last time) by status bars.	The response of the motion assist mechanism showed that hand motion assisted robot has good-to-excellent properties and a high potential for providing hand rehabilitation therapy by self-motion control.
(Grimm et al., 2016)	Upper limb	8-156 months	Fugl-Meyer Motor Assessment (FMA)	Armeo Spring	7	Not available	Shoulder, elbow and wrist joints	Commercial	Arm	Clinical	Virtual Reality	5	4 weeks /20 sessions	20	The level of difficulty for the exercise was adjusted by a performance-dependent real-time adaptation algorith	Feedback as to the movement quality, i.e., the absence or presence of compensatory movements was provided.	The subjects received brain self-regulation and proprioceptive feedback and closed-loop virtual reality feedback from their three-dimensional movements.	There was an improvement of kinematic parameters and therefore was significant motor improvements in grip force and elbow movement.

															m			
(Forrester et al., 2011)	Lower limb	> 6 months	Robot-based measures to evaluate ankle impairment and gait function	Ankle bot	3	Not available	Ankle	Commercial	Ankle	Clinical	Video games	8	6 weeks /3 times weekly	60 minutes	Easy-to-difficult	Not mentioned	Visual feedback was presented during movement	Improved paretic ankle motor control was seen as increased target success, along with faster and smoother movements. Walking velocity also increased significantly, whereas durations of paretic single support increased, and double support decreased.
(Mirelman et al., 2010)	Lower limb	> 2 years	Two factorial (training regime time) repeated measures analysis of variance	Rutgers ankle rehabilitation system (RARS)	6	Not available	Foot	Commercial	Foot	Clinical	Virtual Reality	18	4 weeks /3 times per week	60 minutes	Not applicable	Not applicable	Feedback is provided with VR - visual and auditory feedback	The ankle profiles of the affected side showed an increase in the peak plantar flexion moment, and ankle power generation.
(Comani et al., 2015)	Upper limb	sub-acute stage	Motricity Index/Barthel Index	Trackhold	Not mentioned	Motor	Arm	Commercial	Arm	Clinical	Virtual Reality	3	13 sessions	-	Not applicable	Not applicable	Feedback is provided with VR - visual and auditory feedback	The patterns of activation with specific features and recovery trajectories for each patient was observed and improvements were observed.
(Connelly et al., 2010)	Upper limb	-	Fugl-Meyer Motor Assessment (FMA)	PneuGlove	6	Servo motor	Hand	Commercial	Hand	Clinical	Virtual Reality	14	6 Weeks	60 minutes	Not applicable	Not applicable	Wide5 Head Mounted Display (HMD) is used as a display device to provide a measure of haptic feedback.	Significant increases in the upper extremity Fugl-Meyer Assessment scores were achieved.

(Delbressine et al., 2012)	Upper limb	-	Not mentioned	Sensor Jacket	Not mentioned	-	Chest and Shoulder	Prototype	Chest	Clinical	Games	12	NA	40 minutes	Difficulty level was changing as per participants.	Real time is provided through a graphical representation consisting of two bars, one for the shoulder and one for trunk movement.	Vibrotactile feedback is provided on the corresponding body part on a graphical interface.	Need more improvement in choosing the right game for patients.
(Gijbels et al., 2011)	Upper limb	27 ± 10 years	TEMPA	Armeo Spring	5	Non robotic actuator / Passive system	Arm	Commercial	Arm	Clinical	Virtual Reality	10	8 weeks / 3 times per week	30 minutes	Not applicable	Not applicable	Auditory and visual performance feedback during and after practice	TEMPA scores improved (p = 0.02), while a trend towards significance was found for the 9HPT (p = 0.05)
(House et al., 2016)	Upper limb	Average of 98 (45) months	Fugl-Meyer Motor Assessment (FMA)	Bright Arm Duo Arm Support	Not mentioned	Electrical actuators	Arm	Commercial	Arm	Clinical	Games	7	8 weeks	20 min - 50 min	Games	Nine games included summative performance feedback which provided positive reinforcement and were meant as morale boosters.	Feedback of the table current status was made available to the therapist at the laptop station or output display	The most significant improvement was in grasp strength of the affected arm from an average of 7.6 N pretherapy to 17.2 N post-therapy
(Buschfort et al., 2010)	Upper limb	4.3 weeks	Fugl-Meyer-Assessment and Action Research Arm Test	Arm studio	Not mentioned	Not available	Arm	Commercial	Arm	Clinical	Games	24	3 weeks /15 sessions	30-45 min	Games	Not mentioned	Computer biofeedback, via a wireless mouse connected to the combining rod, is optional.	The arm studio provides high-intensity impairment oriented training may have great potential for the rehabilitation of the upper limb of severely affected patients after stroke in terms of cost effectiveness and customizable effects.
(Cameirao et al., 2012)	Upper limb	1334 ± 297 days	Barthel Index, Motricity Index22 , Modified Ashworth Scale, Fugl-Meyer Assessm	RGS-Haptic (RGS-H)	6	Not available	Arm	Prototype	Arm	Clinical	Virtual Reality	14	5 weeks (4 days a week)	35 min	Difficulty level was updated online by a psychometrically validat	Not applicable	Visual feedback was presented during movement	Improvements were assessed more in patients allocated to the RGS-H group (average rating of 3.7 against 2.8 for RGS and 2.9 for RGS-E).

			ent, Chedoke Arm and Hand Activity Inventory (CAHAI), Nine Hole Peg Test and Box and Block Test.												ed adaptive controller, or Personalized Training Module			
(da Silva Cameirão et al., 2011)	Upper limb	3 weeks	Fugl-Meyer Motor Assessment (FMA)	Data Gloves	Not mentioned	Not available	Hand	Prototype	Hand	Clinical	Virtual Reality	8	12 weeks	20 min	New difficulty setting was computed taking into account the previous responses of the user	Not applicable	Sensory feedback of one's actions in terms of movement patterns and movement outcomes was provided.	Rehabilitation Gaming System speeds-up the recovery of the deficits of the upper extremities, with particular emphasis on functional aspects related to the performance of the activities of daily living.
(Liao et al., 2012)	Upper limb	23.90 ± 13.39 months	Fugl-Meyer Motor Assessment (FMA)	Bi-Manu-Track	Not mentioned	Not available	Arm	Not mentioned	Arm	Clinical	Games	10	20 sessions/ 4 weeks (5 days a week)	90-105 minutes a day	Not mentioned	Real-time visual feedback about the actions or force.	Variety of variety of sensorimotor feedback such as visual and auditory feedback during training sessions is provided.	The robot-assisted therapy group handled more daily tasks with the impaired arm than the control group. Also motor function was greatly improved.
(Hsieh et al., 2011)	Upper limb	21.33 (7.17) months for Group 1 and 13.	Fugl-Meyer Motor Assessment (FMA)	Bi-Manu-Track	Not mentioned	Not available	Arm	Not mentioned	Arm	Clinical	Games	6	4 weeks (5 days a week)	90-105 min/d,	Not mentioned	Visual movement feedback was provided from the game	The therapist also provided the verbal feedback with the visual feedback from the game.	Post hoc analyses revealed that the higher intensity RT group showed significantly better improvements in motor ability than the lower intensity RT group (P = .04)

		00 (7.04) for Group 2																
(Zhang et al., 2011)	Upper limb	-	Wolf Motor Function Test (WMFT) and Fugl Meyer Assessment (FMA).	RUPERT	5	Pneumatic (PAMs)	Arm	Commercial	Arm and Shoulder	Home	Virtual Reality	2	once/weekday for 4 weeks	45	Changed by therapist.	Not applicable	-	Out of 2 patients, 1 showed improvements in movement smoothness on targets whereas second patient did not experience ascending nor descending trend in smoothness.
(Frisoli et al., 2012)	Upper limb	Between 3 and 9 years	Fugl-Meyer Assessment scale and Modified Ashworth scale	L-Exos	4	Electric motors	Arm	Commercial	Arm	Clinical	Virtual Reality	9	18 sessions/6 weeks	60 minutes	Not applicable	Not applicable	-	There was an improvement of the motor performance and the reduction of the muscular spasticity.
(Mihelj et al., 2012)	Upper limb	128 ± 64 days	-	Haptic Master robot	3	DC brushed motors	Arm	Commercial	Arm	Clinical	Virtual Reality (Games)	16	Twice a week/ 2 weeks	Minimum 5 minutes	Adjusted by therapist	Not applicable	Visual and Acoustic feedback is provided.	Helps in increasing motivation of patients in rehabilitation process.
(Sivan et al., 2014)	Upper limb	24.8 (17.8) months	Optotrak kinematic variables, Fugl Meyer Upper Extremity motor subscale (FM-UE), Action Research Arm Test (ARAT), Medical Research Council (MRC)	Home-based Computer Assisted Arm Rehabilitation (hCAAR) robotic device	-	-	Hand	-	Hand	Home	Games	17	8 weeks	Mean device usage time 520 min	Level was changing as per the extent of workspace used.	Not mentioned	Visual and auditory feedback of the target location and the movement of the joystick was provided.	The percentage improvement in the median movement time at A1 was by 19%, path length improved by 15% and jerk improved by 19%

			and Modified Ashworth Scale (MAS), Chedoke Arm and Hand Activity Inventory (CAHAI) and ABILHAND															
(Amirabdollahian et al., 2014)	Upper limb	11 months (± 6 months).	Fugl-Meyer Motor Assessment (FMA)	SCRIPT Prototype 1 (SP1)	-	-	Hand (thumb and finger)	Prototype	Hand	Home	Games	12	6 Weeks	30 min per day,	Game difficulty increases when lateral movement of the arm are added to the flexion. and extension of the hand	Not mentioned	(Bio) feedback is provided about patient's performance	The study showed the feasibility of independent home-based training with the SCRIPT system.
(Stein et al., 2011)	Upper limb	> 6 months	Fugl-Meyer Motor Assessment (FMA)	Amadeo	-	Not mentioned	Fingers and Hand	-	Hand	Clinical	Games	12	6 Weeks /3 days per week	1 hour	Changed as per user.	Visual feedback was provided using two games.	Not mentioned	Improvements were found in multiple measures of motor performance, including the Upper Extremity Fugl-Meyer, the Motor Activity Log, the Manual Ability Measure-36, and the Jebsen Hand Function Test.
(Dovat et al., 2010)	Upper limb	2 years to 6 years	Fugl-Meyer Motor Assessment (FMA)	HandCARE	Robotic device	DC brushed motors	Hand and Fingers	Not mentioned	Fingers	Clinical	Games	2	8 weeks (two sessions per week)	20 min	Not mentioned	Games were based on visual feedback and provided virtual reality environments.	Used multi-sensory feedback to increase motivation.	There was a noticeable improvement in the Chedocke-McMaster impairment inventory which illustrated the potential of the HandCARE exercises and validated the use of the proposed metrics to evaluate hand and finger function.

(Novak et al., 2014)	Upper limb	2 years to 21 years	Fugl-Meyer Motor Assessment (FMA)	ARMin IV	7	-	Arm	Commercial	Arm and forearm	Clinical	Games	30 unimpaired and 8 impaired	Once	Three 5-min rounds	Game difficulty was varied during the practice round and finally set at a level appropriate for subjects	-	Feedback was taken from participants using questionnaires.	Study showed that stroke patients prefer playing two-player rehabilitation game as compared to single-player one, as they enjoy being able to talk and otherwise interact with the other person.
(Merians et al., 2010)	Upper limb	6 (5) years	Fugl-Meyer Motor Assessment (FMA)	Cyber Glove	-	-	Hand	Commercial	Hand	Clinical	Virtual Reality (Games)	11	2 weeks	2-3 Hour/session	Not mentioned		Visual and auditory feedback	Limb control was effective and there were robust changes in functional tests of upper extremity motor control, the Wolf Motor Function Test, the Jebsen Test of Hand Function and the 9-hole Peg Test.
(Kan et al., 2011)	Upper limb	227 days	Chedoke-McMaster Stroke Assessment (CMSA)	Robotic device	-	-	Trunk	Not mentioned	-	Clinical	Virtual Reality	1		Three times a week for two weeks	Not applicable	Visual feedback on hand position and target location	Feedback was taken from participants using questionnaires.	POMDPs (a partially observable Markov decision process) have promising potential to provide autonomous upper-limb rehabilitation for stroke patients.
(Simkins et al., 2012)	Upper limb	> 6 months	Chedoke-McMaster Stroke Assessment (CMSA)	EXO	Not mentioned	-	Arm	Commercial	Arm	Clinical	Games	10	12 sessions	90 min	-	Not mentioned	Feedback was taken from participants using questionnaires.	Subjects preferred static games to dynamic games when attending sessions for post-rehabilitation with the help of an exoskeleton.
